# Analysis of FGF-Dependent and FGF-Independent Pathways in Otic Placode Induction

**DOI:** 10.1371/journal.pone.0055011

**Published:** 2013-01-23

**Authors:** Lu Yang, Paul O’Neill, Kareen Martin, Juan C. Maass, Vassil Vassilev, Raj Ladher, Andrew K. Groves

**Affiliations:** 1 Departments of Neuroscience and Molecular and Human Genetics, Baylor College of Medicine, Houston, Texas, United States of America; 2 Program in Developmental Biology, Baylor College of Medicine, Houston, Texas, United States of America; 3 Division of Cell Biology and Genetics, House Research Institute, Los Angeles, California, United States of America; 4 RIKEN Center for Developmental Biology, Chuo-ku, Kobe, Japan; 5 Department of Otolaryngology, Hospital Clínico Universidad de Chile, Santiago, Chile; 6 Department of Otolaryngology, Clínica Alemana de Santiago, Facultad de Medicina Clínica Alemana-Universidad del Desarrollo, Santiago, Chile; Texas A&M University, United States of America

## Abstract

The inner ear develops from a patch of thickened cranial ectoderm adjacent to the hindbrain called the otic placode. Studies in a number of vertebrate species suggest that the initial steps in induction of the otic placode are regulated by members of the Fibroblast Growth Factor (FGF) family, and that inhibition of FGF signaling can prevent otic placode formation. To better understand the genetic pathways activated by FGF signaling during otic placode induction, we performed microarray experiments to estimate the proportion of chicken otic placode genes that can be up-regulated by the FGF pathway in a simple culture model of otic placode induction. Surprisingly, we find that FGF is only sufficient to induce about 15% of chick otic placode-specific genes in our experimental system. However, pharmacological blockade of the FGF pathway in cultured chick embryos showed that although FGF signaling was not sufficient to induce the majority of otic placode-specific genes, it was still necessary for their expression in vivo. These inhibitor experiments further suggest that the early steps in otic placode induction regulated by FGF signaling occur through the MAP kinase pathway. Although our work suggests that FGF signaling is necessary for otic placode induction, it demonstrates that other unidentified signaling pathways are required to co-operate with FGF signaling to induce the full otic placode program.

## Introduction

The entire inner ear and the neurons that innervate it are derived from the otic placode, a patch of thickened ectoderm that lies on either side of the posterior hindbrain [1], [2], [3]. The otic placode, together with the nasal, lens, trigeminal and epibranchial placodes derive from a circumferential band of ectoderm running around the anterior neural plate. This “pre-placodal” domain is molecularly distinct from the neural plate, epidermis and emerging neural crest, and is induced and positioned by a combination of activating and inhibitory signals from the neural plate, epidermis and underlying mesendoderm [4], [5], [6]. The pre-placodal domain gives rise to groups of placodal progenitor cells that are initially intermingled [7], [8], [9], but later become distinct and regionally restricted in response to local inducing signals. It is now well-established from studies in all major vertebrate groups that FGF signaling is necessary to initiate the induction of the otic placode. The source and identity of the inducing FGF family members vary between different vertebrate species – for example, *Fgf3* and *Fgf8* present in the hindbrain co-operate to induce the otic placode in zebrafish [10], [11], [12], whereas in mice, hindbrain-derived *Fgf3* and mesodermally-derived *Fgf10* are necessary for otic placode induction [13], and in chickens, both *Fgf3* and *Fgf19* are initially expressed in mesoderm underlying the presumptive otic placode and later become expressed in the hindbrain adjacent to the otic placode [14], [15], [16].

The induction of the otic placode in response to FGF signaling proceeds in a series of steps [5], [17], some of which can be experimentally uncoupled [18], [19], [20]. The first evidence of regional differentiation within the posterior pre-placodal domain is the expression of the transcription factors *Pax2* and *Pax8* [11], [12], [19], [21]. Fate-mapping of this region using DiI in chick embryos or Pax2-Cre transgenic mice shows that, in addition to giving rise to the otic placode, *Pax2*-expressing cells will also contribute to both epidermis and at least some of the epibranchial placodes [5], [8], [22], [23]. This *Pax2*-expressing progenitor domain has been termed the pre-otic field or the otic-epibranchial progenitor domain (OEPD) [2], [5], [17], [24]. The decision to become otic versus non-otic derivatives is mediated in part by the duration of FGF signaling in the OEPD, with transient FGF signaling tending to favor otic differentiation, while sustained FGF signaling appears to repress otic differentiation and permit differentiation of the epibranchial placodes [24]. Consistent with this idea, some of the first genes to be up-regulated in the otic placode are negative regulators of FGF/MAP kinase signaling such as members of the *Sprouty* and *Dusp* dual-specificity phosphatase families [25], [26], [27]. Wnt signaling, emanating from the midline and neural folds also acts to distinguish otic progenitors from their neighbors in the OEPD. High levels of Wnt signaling direct OEPD progenitors towards an otic fate, whereas reducing or blocking Wnt signaling greatly reduces the size of the otic placode and expands surrounding epidermis [5], [17], [24], [28].

The signals that direct the subsequent differentiation and development of the otic placode after establishment of the *Pax2* ^+^ OEPD are far less well-characterized. Although FGF signaling is clearly necessary for otic placode induction, it is not clear whether it is sufficient to induce the otic placode, or whether other signals also play a role in the induction of otic markers. At one extreme, FGF signaling might simply initiate the induction of the very first otic genes such as *Pax2*, with other signals from the hindbrain and mesoderm inducing other otic genes sequentially or in parallel with FGFs. Alternatively, FGF signaling might be sufficient for the induction of most otic placode genes. Although recent microarray studies have identified a number of otic placode genes that require FGF signaling for their induction [29], the sufficiency of FGF in otic induction has only been examined for a very small number of genes, including *Pax2* [30].

In an attempt to better distinguish between the necessity and sufficiency of FGF signaling in otic placode induction, we sought to estimate the proportion of otic placode genes that can be induced by exposure to FGFs. We assembled a list of otic-specific genes by carrying out a microarray comparison of otic versus non-otic ectoderm in the chick embryo. We then compared this with a list of FGF-responsive genes obtained using microarrays to identify genes induced by culturing pre-placodal ectoderm in FGF2. We were surprised to find that out of 345 otic-specific transcripts identified in our microarray experiments, only 52 were also induced by FGF signaling in culture, suggesting that FGF signaling is only sufficient to induce a small proportion of otic placode genes. We used pharmacological inhibitors of the FGF pathway in chick embryos to show that although FGF signaling was not sufficient to induce the majority of otic-specific genes, it was still necessary for their expression in vivo. Our inhibitor experiments also suggest that the early steps in otic placode induction regulated by FGF signaling likely occur through the MAP kinase pathway. Our work suggests that while FGF signaling is clearly necessary for otic placode induction, other currently unidentified signaling pathways may be required to co-operate with FGF signaling to induce the majority of otic placode genes.

## Materials and Methods

### Chick Embryos

Fertilized chicken eggs were obtained from local commercial suppliers (AA Labs, Westminster, CA or Ideal Poultry, Cameron, TX) and incubated in a humidified atmosphere at 37.8°C. Embryos were staged according to the number of somite pairs (ss) or by the Hamburger and Hamilton (HH) staging system [31].

### Floating Chick Embryo Cultures

“Cornish pasty” embryo cultures were made with minor modifications to the methods of Nagai and colleagues [32]. Stage HH5-8 embryos were dissected from eggs and excess yolk was removed. The embryos were folded in half along the midline into a semi-circle, and cut along the outside edge. A series of small cuts were made along the round edge of the semicircular blastoderm to ensure proper sealing. The embryos were allowed to rest undisturbed in Ringer’s solution at room temperature for approximately 30 minutes and then transferred to 6-well plates containing 3 ml of medium (a 2∶1 mixture of thin albumen and Ringer’s solution with penicillin and streptomycin). Each well contained no more than five embryos. The cultures were grown at 37.8°C for 24 hours, to approximately HH stage 11–12. The embryos were then dissected free of the surrounding area opaca and fixed in 4% paraformaldehyde overnight at 4°C.

### Chick Electroporation

Chick embryos at HH stages 6–7 were explanted as “EC” cultures on filter paper and maintained on agar-albumen plates as described in [33]. Embryos were electroporated with a dominant-negative mutation of rat MEK-1 containing two point mutations (S218A and S222A) [34] cloned into the pCIG vector that contains an IRES-GFP sequence, or with pCIG alone. DNA was diluted at 3 µg/µl in Howard’s Ringer solution containing 0.1% Fast Green and applied using a Picospritzer II pressure injector. Embryos received five 50 ms pulses of 15 V each, delivered through a custom-built square-wave electroporator. Electroporated embryos were then returned to their agar-albumen plates and cultured until they had reached at least HH stage 10 before being fixed and processed for in situ hybridization for *Pax2* and immunohistochemistry for GFP.

### Collagen Gel Ectoderm Cultures

**Figure 1:**
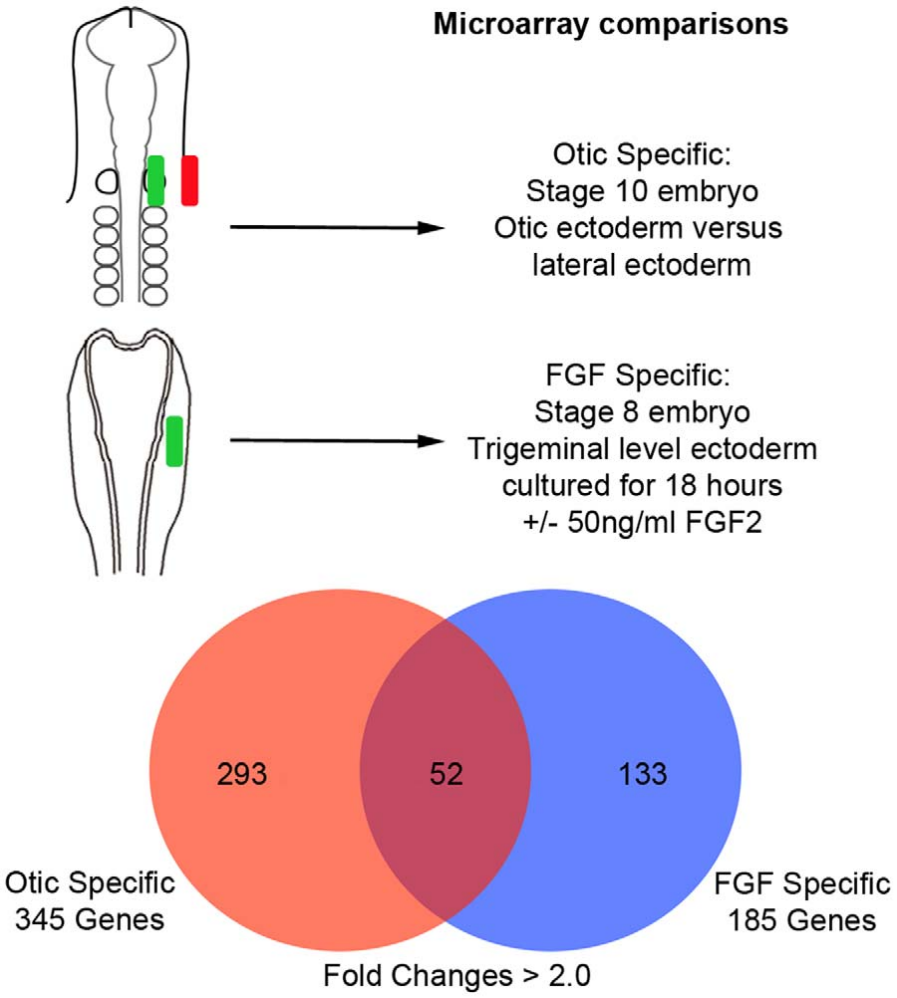
Design of the microarray experiments used in the paper. In the first set of comparisons, otic placode tissue (green) and non-otic tissue lateral to the otic placode (red) were dissected from Hamburger and Hamilton stage 10 chick embryos and gene expression compared by Affymetrix microarrays. In the second set of comparisons, presumptive trigeminal placode ectoderm was dissected from Hamburger and Hamilton stage 8 chick embryos and cultured in collagen gels in the presence or absence of 50 ng/ml FGF2 for 18 hours. Gene expression in the FGF2-treated and control samples was again compared by Affymetrix microarrays. The Venn diagram shows genes that were significantly (p<0.05) up-regulated in each experiment.

0–4 ss embryos (HH stage 6–8) were dissected from eggs, washed with Ringer’s solution and treated with 0.1 mg/ml dispase in DMEM/F12 medium on ice for 15 minutes, then at 37°C for 10 minutes. Digestion was stopped by washing the embryos with 10% fetal bovine serum in DMEM for 10 minutes on ice and keeping them on ice in Ringer’s solution. Using 30-gauge hypodermic needles, prospective trigeminal, otic level or lateral ectoderm was isolated from embryos (see Figure 1) and held on ice in Ringer’s solution until needed.

As described by Groves and Bronner-Fraser [19], collagen matrix gels were prepared by mixing 10 parts bovine collagen solution with 1 part 10×MEM. The solution was brought to neutral pH by adding 7.5% sodium bicarbonate drop-wise, typically to a final concentration of 0.35%. Ten to fifteen pieces of ectoderm were suspended in a 50 µl drop of the collagen mixture, and allowed to solidify at 37°C for 20 minutes. Each collagen matrix gel was cultured in 500 µl of DMEM-BS medium (a modification of the chemically defined medium of [35], [36]) and growth factors or inhibitors were added as appropriate. Explants were cultured at 37°C for 18–24 hours. Excess collagen was removed and explants were rinsed with PBS for RNA extraction. In some cases, manually dissected chunks of head tissue including the neural tube, mesoderm and endoderm were also cultured in collagen gels.

### Activation and Pharmacological Inhibition of the FGF Pathway

Recombinant human FGF2 was obtained from the NCI Biological Resources Branch and prepared as a 10 µg/ml stock in L15 medium containing 0.5% BSA. The following inhibitors were used in this study: SU5402 (FGF receptor inhibitor; 0.5–20 µM); U0126 (MEK inhibitor; 1–40 µM); SB203580 (p38 MAP kinase inhibitor; 1–100 µM), LY294002 (PI3 kinase inhibitor; 1–40 µM), Wortmannin (PI3 kinase inhibitor; 0.1–10 µM), U73122 (PLCgamma inhibitor; 1–100 µM) and AKT inhibitor IV (1–20 µM). All inhibitors were prepared as stock solutions in DMSO and stored in the dark at −20°C. In all experiments involving these inhibitors, DMSO was added to control cultures at the same concentration as the cultures containing inhibitors.

### Probe Synthesis and in situ Hybridization

Digoxygenin-labeled, cRNA probes were synthesized for whole mount in situ hybridization according to the protocol of Stern [37], using plasmid clones from the following sources: Brian Huston (*BMP7*), Doug Engel (*Gata3*), Berta Alsina (*Lmx1b*), Domingos Henrique (*Pax2*), and Jonathan Raper (*Robo2*). Chicken EST clones from the BBSRC Chick EST Database [38] were used to generate probes for *Cyp26C1* (ChEST102p18), *FGF8* (ChEST438h15) and *Foxi2* (ChEST912m14). Probes for *Foxg1, Has2,* and *Sox8* were generated by transcription of amplified PCR products from chick genomic DNA. A T7 polymerase sequence (GGATCCTAATACGACTCACTATAGGGAG) was added to the 5′ end of the reverse primer for each target gene. The primer sequences used to generate PCR probes are listed in Table S4. PCR products were purified using QIAquick PCR Purification Kit (Qiagen). *NGFR* and *SMOC1* probes were transcribed from the corresponding Affymetrix consensus sequences cloned from chick cDNA. Detailed protocols for probe synthesis and whole mount in situ hybridization are available from the corresponding authors.

### Immunocytochemistry

For cryosections, embryos were equilibrated in 15% sucrose in PBS, embedded in gelatin (7.5% gelatin, 15% sucrose in PBS) and frozen in liquid nitrogen. 18 µm thick sections were collected on Superfrost Plus slides and stored at −20°C. Slides were placed in PBS at 50°C for 15 minutes to remove gelatin, washed twice in PBS containing 0.1% Triton X-100 and blocked in PBS containing 0.1% Triton X-100 and 5% goat serum for 2–3 hours. Primary antibodies were diluted in blocking buffer and applied overnight at 4°C. The slides were washed twice in PBS/0.1% Triton X-100 and secondary antibodies were diluted in blocking solution and applied for 45 minutes at room temperature. Slides were washed twice in PBS/0.1% Triton X-100 and incubated in DAPI solution for 10 minutes, then washed in PBS before being mounted in Fluoromount G (Southern Biotechnology). Antibodies used in this study were a rabbit polyclonal antibody to Pax2 (Invitrogen), a rabbit polyclonal antibody to GFP (Invitrogen) and a rabbit polyclonal antibody to phosphorylated p44/p42 MAP kinase (Cell Signaling). Whole mount immunocytochemistry for Pax2 and GFP was performed using the same blocking and washing buffers. Whole mount phospho-MAP kinase staining was performed according to a protocol from Janet Rossant [39].

### RNA Extraction, cDNA Synthesis and Real-Time PCR

RNA was extracted from the explants using a Qiagen RNeasy Plus Micro Kit according to the manufacturer’s protocol. RNA was reverse transcribed using SuperScript™ III First-Strand Synthesis System for RT-PCR (Invitrogen) with random hexamers. The resulting cDNA was stored at −20°C until needed. Quantitative PCR was used to analyze the relative abundance of *Auts2*, *Bmp7*, *Foxg1*, *Gata3*, *Has2*, *Lmx1b*, *Pax2*, *Robo2*, *Sox8, Elk3, EphA4* and *Spry2* using a Step One Plus Real-Time PCR System with SYBR® Green Reagents (Applied Biosystems). Chick GAPDH was used as a reference housekeeping gene. The primer sequences used are listed in Table S5. All samples were run in triplicate and included negative controls. A minimum of four independent experiments were analyzed for each gene in each condition. Relative gene expression was analyzed by using the 2^−ΔΔCT^ method [40] and compared for significance with a Mann-Whitney test.

### Microarray Analysis

Otic or lateral ectoderm was isolated from embryos with between 9–11 pairs of somites using enzymatic and manual tissue separation as described above. Trigeminal ectoderm was isolated from embryos with between 0–4 pairs of somites, and cultured in collagen gels in the presence or absence of 50 ng/ml FGF2 for 18 hours. In all cases, ectoderm samples were flash frozen in liquid nitrogen before RNA isolation. Approximately 80 ectoderm samples were used for each replicate, with two replicates being used for each experimental condition.

Cells lysis was performed using QIAshredder columns (Qiagen) and RNA isolated using the RNeasy Micro kits (Qiagen) according to the manufacturer’s protocols, although the DNAseI step was omitted and an additional RNA cleanup step was employed. RNA was amplified and biotinylated using the Two-Cycle cDNA synthesis kit and IVT labeling kit (Affymetrix), before being hybridized with GeneChip Chicken Genome microarrays (Affymetrix). Differential gene expression was analyzed using Bioconductor’s Limma package (www.bioconductor.org) and probes ranked by fold up-regulation using Excel. A cut off value of 2-fold up-regulation was selected to generate the otic and FGF-specific gene sets. Duplicates were manually removed from the final tables; in these cases (indicated by *) the fold-up-regulation listed represents the highest of the replicated probe set values. Microarray data has been deposited in the NCBI GEO database, Accession Number GSE42845.

## Results

### FGF Signaling is Sufficient to Induce only a Subset of Otic Placode Genes in Pre-placodal Ectoderm

A previous study by Mansour and colleagues used microarrays to examine the necessity of FGF signaling in otic placode induction by comparing wild type otic tissue to tissue obtained from *FGF3/10* double knockout mouse embryos [29]. We took a complementary approach by examining the sufficiency of FGF signaling in otic placode induction. We first performed a microarray experiment to identify genes enriched in the chick otic placode shortly after induction. We isolated pieces of otic placode ectoderm from stage 9–10 chicken embryos, using the known expression pattern of *Pax2* at this stage as a landmark for the otic placode [19]. We used Affymetrix microarrays to compare the transcriptional profile of otic placode ectoderm with more lateral ectoderm from the same axial level that includes presumptive epidermis and some progenitors for the epibranchial placodes (Figure 1). We found 345 transcripts that were enriched in otic ectoderm versus lateral ectoderm (>2 fold difference). To determine how many otic placode genes could be induced by FGF signaling alone, we performed a second microarray experiment in which we cultured presumptive trigeminal ectoderm from stage 7–8 chick embryos (1–4 somite pairs) in collagen gels for 18 hours in the presence or absence of 50 ng/ml FGF2. We chose presumptive trigeminal ectoderm for two reasons – first, we previously showed that non-otic regions of the pre-placodal domain, such as the presumptive trigeminal placode, are competent to induce at least some otic placode markers in response to FGFs [30]. Second, by taking presumptive trigeminal ectoderm we reduced the possibility of contamination of our samples by early otic placode transcripts. We found 185 transcripts that were enriched in samples cultured in FGF2 versus those cultured without FGF2 (>2 fold enrichment).

**Figure 2:**
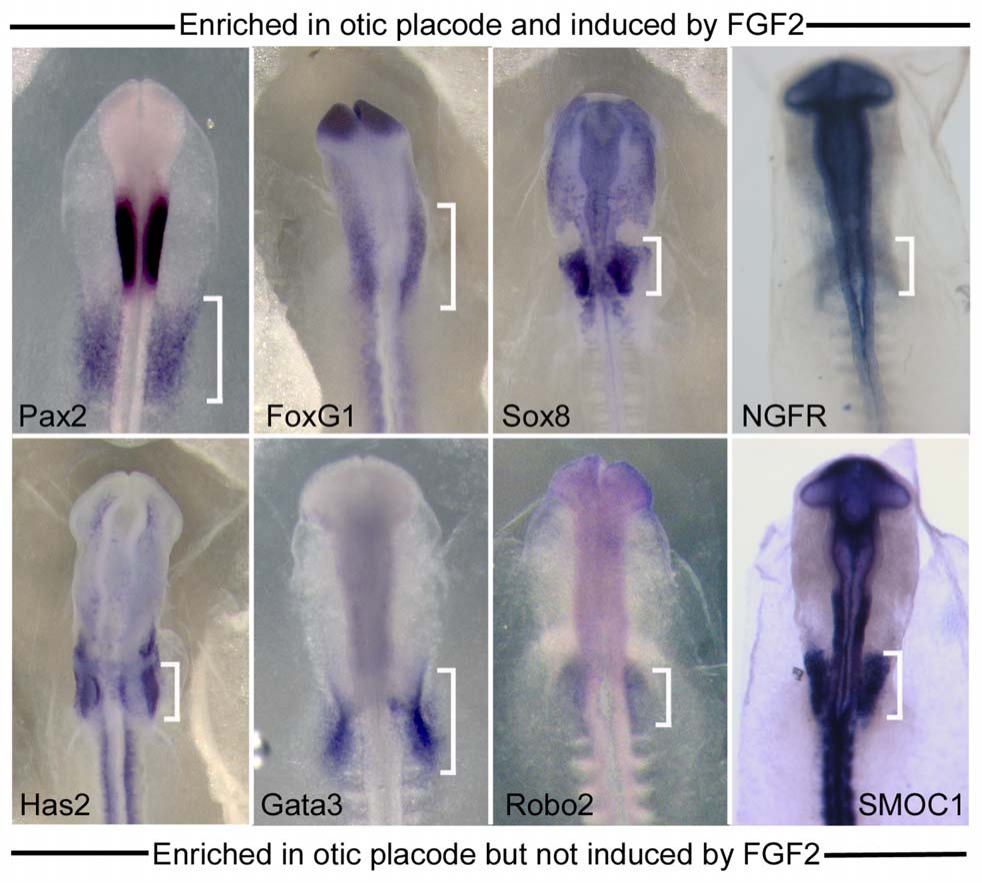
Validation of genes enriched in the microarray experiments by in situ hybridization. The upper panel shows expression of four sample genes (*Pax2, Foxg1, Sox8* and *NGFR*) that were up-regulated in both the otic placode and FGF-treated microarray experiments. The lower panel shows four sample genes (*Has2, Gata3, Robo2* and *SMOC1*) that were enriched in the otic placode but were not induced by FGF treatment. The otic region is indicated with white brackets.

The validity of our data was supported by the presence of many well-characterized transcripts previously reported to be expressed in the otic placode, or associated with the FGF signaling pathway. The top 20 enriched genes are shown in Table 1 (otic genes), Table 2 (genes up-regulated by FGF), Table 3 (otic genes that are also up-regulated by FGF) and Table 4 (otic genes that are not up-regulated by FGF). Full gene lists are provided in Tables S1, S2, S3. Otic placode genes identified in our microarrays included *Pax2*, the earliest known marker of the otic placode in chick [19], and the otic placode-associated transcription factors *GATA3*, *Tbx1*, *Gbx2, Foxg1*, *Sox2* and *Sox8* [19], [41], [42], [43], [44], [45]. Other general markers of placodal identity were also detected, including *Eya2, Eya4,* and *Dach1* [6]. From the list of genes enriched in both otic placode and FGF treatment groups, we examined the expression of *Pax2*, *FoxG1*, *Sox8* and *NGFR* by in situ hybridization (Figure 2). As expected, these transcripts were all detected in the otic placode, however the expression of *FoxG1* and *NGFR* was not restricted to the otic region, with the signal extending into anterior head ectoderm. Similarly we looked at the expression of *Has2*, *GATA3*, *Robo2*, and *SMOC1*, genes predicted to be present in the otic placode, but which are not FGF-induced. These transcripts were all detected in the otic ectoderm (Figure 2). This suggests that our array approach is reliable in detecting otic placode enriched factors. However, it is also likely that our data set includes genes present in the otic placode plus other surrounding tissues, and does not represent a pure population of truly otic specific genes.

**Table 1:** Top 20 Otic-enriched genes.

ID	Symbol	Name	FGF Regulated?	Fold Up-regulation	Notes and references
Gga.1710.1.S1_at	Hs3st3b1	heparan sulfate glucosamine 3-O-sulfotransferase 3B1	YES	32.1	Unknown
Gga.2354.1.S1_at	SOX8	SRY (sex determining region Y)-box 8	YES	25.0	Expressed in chick otic placode [62], [64]
Gga.19393.1.S1_s_at	CYP26C1	cytochrome P450, family 26, subfamily C, polypeptide 1	YES	14.5	Not known to be expressed in otic region
Gga.17119.1.S1_at	Prdm12	PR domain zinc finger protein 12	NO	11.5	Unknown
Gga.7581.1.S1_at	SOX2	SRY (sex determining region Y)-box 2	NO	11.3	Expressed in chick otic placode [65], [66]
Gga.1839.1.S1_at	EYA2	eyes absent homolog 2 (Drosophila)	YES	11.2	Expressed weakly in otic in chick [46]
Gga.10.1.S1_at	OTX2	orthodenticle homeobox 2	NO	10.9	Not known to be expressed in otic region
Gga.19378.1.S1_at	IL17RD	interleukin 17 receptor D	YES	10.8	Expressed in chick otic placode [67]
Gga.205.1.S1_at	FGF19	fibroblast growth factor 19	NO	9.8	Expressed in mesoderm and endoderm [57], [68]
Gga.3615.1.S2_at	FST	follistatin	NO	9.6	Expressed in mesoderm
Gga.565.1.S1_at	GBX2	gastrulation brain homeobox 2	YES	9.6	Expressed in chick otic placode [54]
Gga.6245.2.S1_at	NGFR	nerve growth factor receptor (TNFR superfamily, member 16)	YES	9.4	Expressed in otocyst of chick and rat [69]
Gga.469.2.A1_at	FOXC2	forkhead box C2 (MFH-1, mesenchyme forkhead 1)	NO	9.1	Not known to be expressed in otic region
Gga.12157.1.S1_at	PKDCC	protein kinase domain containing, cytoplasmic homolog	NO	8.4	Not known to be expressed in otic region
Gga.5787.1.S1_at	SMOC1	SPARC related modular calcium binding 1	NO	7.8	Expressd in chick otic placode (this study)
Gga.322.1.S1_at	SPRY1	sprouty 1	YES	7.6	Expressed in head ectoderm [26]
GgaAffx.20987.1.S1_at	PAX2	Pax2 paired box gene 2	YES	7.3	Expressed in otic placode in chick [19]
Gga.15383.1.S1_at	CCDC3	coiled-coil domain containing 3	NO	7.3	Not known to be expressed in otic region
Gga.2422.1.S1_at	ENS-3	pol-like protein ENS-3	YES	7.1	No expression data
Gga.3219.1.S1_at	FIGF	c-fos induced growth factor (vascular endothelial growth factor D)	YES	7.1	Not known to be expressed in otic region

**Table 2:** Top 20 FGF up-regulated genes.

ID	Symbol	Name	Otic Enriched?	Fold Up-regulation	Notes and references
Gga.19393.1.S1_s_at	CYP26C1	cytochrome P450, family 26, subfamily C, polypeptide 1	YES	19.8	Induced by FGF signaling in Xenopus [60]
Gga.12383.1.S1_at	CRHBP	corticotropin releasing hormone binding protein	YES	9.8	No reported link with FGF signaling
Gga.8433.1.S1_a_at	PHACTR1	phosphatase and actin regulator 1	NO	9.0	No reported link with FGF signaling
Gga.1840.1.S2_at	NEUROD	Neurogenic differentiation factor 1	NO	8.8	FGF upstream of NeuroD in otic neuroblast differentiation [61]
Gga.8445.1.S1_at	DUSP6	dual specificity phosphatase 6	YES	7.3	Negative regulator of FGF signaling [63]
Gga.322.1.S1_at	SPRY1	sprouty homolog 1, antagonist of FGF signaling (Drosophila)	YES	6.5	Negative regulator of FGF signaling.
Gga.3374.1.S1_at	SPRY2	sprouty homolog 2 (Drosophila)	YES	6.0	Negative regulator of FGF signaling.
Gga.3063.1.S1_at	MYBPC1	myosin binding protein C, slow type	NO	5.8	No reported link with FGF signaling
Gga.1839.1.S1_at	EYA2	eyes absent homolog 2 (Drosophila)	YES	5.0	Induced by FGF8 in chick pre-placodal region [4]
Gga.661.1.S1_at	FGF8	fibroblast growth factor 8 (androgen-induced)	YES	4.8	FGF ligand
Gga.3807.1.S2_s_at	RALDH3	retinaldehyde dehydrogenase 3	NO	4.6	No reported link with FGF signaling
Gga.1479.2.S1_a_at	PTN	pleiotrophin (heparin binding growth factor 8, neurite growth-promoting factor 1)	NO	4.5	No reported link with FGF signaling
Gga.3047.1.S1_at	EPHA5	EPH receptor A5	NO	4.5	No reported link with FGF signaling
Gga.11969.1.S1_at	CYTL1	cytokine-like 1	NO	4.4	No reported link with FGF signaling
Gga.5879.1.S1_at	PDGF	platelet derived growth factor D (PDGFD)	NO	4.4	No reported link with FGF signaling
Gga.2422.1.S1_at	ENS-3	pol-like protein ENS-3	YES	4.4	No reported link with FGF signaling
Gga.4083.1.S1_at	NKX-6.1	homeodomain protein	NO	4.4	No reported link with FGF signaling
GgaAffx.20874.1.S1_at	ARC	activity-regulated cytoskeleton-associated protein	NO	4.3	No reported link with FGF signaling
Gga.5847.1.S1_at	OXT	oxytocin, prepro- (neurophysin I)	NO	4.3	No reported link with FGF signaling
Gga.8807.1.S1_at	MECOM	MDS1 and EVI1 complex locus	YES	4.1	No reported link with FGF signaling

**Table 3:** Top 20 Otic-enriched genes that are also up-regulated by FGF.

ID	Symbol	Name	Fold Up-regulation
Gga.1710.1.S1_at	Hs3st3b1	heparan sulfate glucosamine 3-O-sulfotransferase 3B1	32.1
Gga.2354.1.S1_at	SOX8	SRY (sex determining region Y)-box 8	25.0
Gga.19393.1.S1_s_at	CYP26C1	cytochrome P450, family 26, subfamily C, polypeptide 1	14.5
Gga.1839.1.S1_at	EYA2	eyes absent homolog 2 (Drosophila)	11.2
Gga.19378.1.S1_at	IL17RD	interleukin 17 receptor D	10.8
Gga.565.1.S1_at	GBX2	gastrulation brain homeobox 2	9.6
Gga.6245.2.S1_at	NGFR	nerve growth factor receptor (TNFR superfamily, member 16)	9.4
Gga.322.1.S1_at	SPRY1	sprouty 1	7.6
GgaAffx.20987.1.S1_at	PAX2	Pax2 paired box gene 2	7.3
Gga.2422.1.S1_at	ENS-3	pol-like protein ENS-3	7.1
Gga.3219.1.S1_at	FIGF	c-fos induced growth factor (vascular endothelial growth factor D)	7.1
Gga.19221.1.S1_at	EBF3	early B-cell factor 3	6.0
Gga.1507.1.S2_at	ISL1	ISL LIM homeobox 1	5.8
Gga.7323.1.S1_at	NEDD9	neural precursor cell expressed, developmentally down-regulated 9	5.8
Gga.331.1.S1_at	CYP26A1	cytochrome P450, family 26, subfamily A, polypeptide 1	5.2
Gga.657.1.S1_at	FOXG1	forkhead box G1	4.6
Gga.17706.1.S1_at	AG2	AG2 homolog	4.5
Gga.3374.1.S1_at	SPRY2	sprouty 2	4.3
Gga.14703.1.S1_at	SP8	Sp8 transcription factor	4.2
Gga.661.1.S1_at	FGF8	fibroblast growth factor 8	4.1

**Table 4:** Top 20 Otic-enriched genes that are not up-regulated by FGF.

ID	Symbol	Name	Fold Up-regulation
Gga.17119.1.S1_at	Prdm12	PR domain zinc finger protein 12	11.5
Gga.7581.1.S1_at	SOX2	SRY (sex determining region Y)-box 2	11.3
Gga.10.1.S1_at	OTX2	orthodenticle homeobox 2	10.9
Gga.205.1.S1_at	FGF19	fibroblast growth factor 19	9.8
Gga.3615.1.S2_at	FST	follistatin	9.6
Gga.469.2.A1_at	FOXC2	forkhead box C2 (MFH-1, mesenchyme forkhead 1)	9.1
Gga.12157.1.S1_at	PKDCC	protein kinase domain containing, cytoplasmic homolog	8.4
Gga.5787.1.S1_at	SMOC1	SPARC related modular calcium binding 1	7.8
Gga.15383.1.S1_at	CCDC3	coiled-coil domain containing 3	7.3
Gga.207.1.S1_at	ZIC2	Zic family member 2	6.6
Gga.2699.1.S1_at	IRX1	iroquois homeobox 1	6.6
Gga.744.1.S1_at	GATA3	GATA binding protein 3	6.5
GgaAffx.21693.1.S1_s_at	RNF150	Ring Finger Protein 150	6.4
Gga.2894.1.S1_at	JAG1	jagged 1	6.2
Gga.3039.1.S1_at	CCND1	cyclin D1	5.8
Gga.1817.1.S1_at	SALL1	sal-like 1 (Drosophila)	5.7
Gga.770.1.S1_at	SOHO-1	sensory organ homeobox protein SOHo	5.7
Gga.5109.1.S1_s_at	MYCN	v-myc myelocytomatosis viral related oncogene, neuroblastoma derived	5.4
Gga.13425.1.S1_at	KCNK2	potassium channel, subfamily K, member 2	5.4
Gga.16364.1.S1_at	NAP1L2	nucleosome assembly protein 1-like 2	5.3

**Figure 3:**
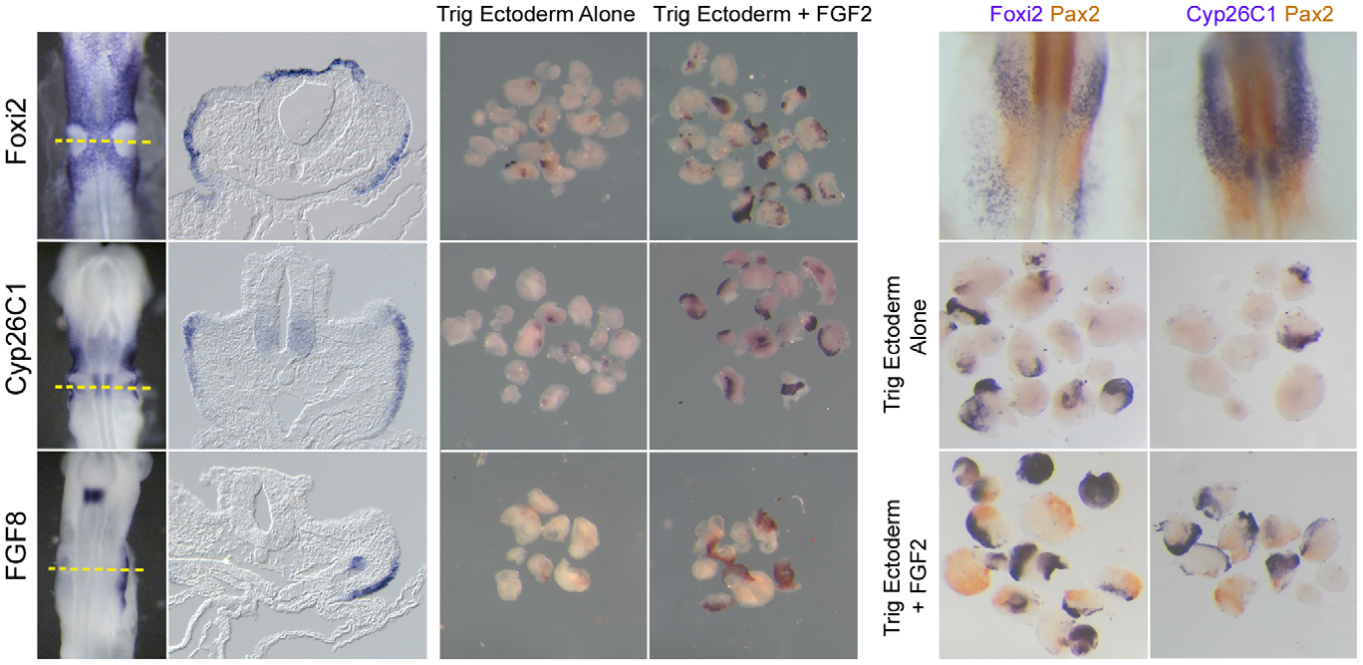
Examples of two genes in our study – *FGF8* and *Cyp26C1*– that were identified as FGF-responsive otic placode genes, but which were shown not to be expressed in the otic placode by in situ hybridization. The left panels show the expression of these genes in non-otic ectoderm compared with *Foxi2*, a gene previously shown to be restricted to non-otic ectoderm [24], [28]. The center panels confirm that all three genes can be up-regulated in collagen gel cultures of presumptive trigeminal ectoderm cultured in 50ng/ml FGF2. The right panels show that the expression of *Foxi2* and *Cyp26C1* (purple label) in such FGF-induced cultures is mutually exclusive with expression of the otic marker *Pax2* (orange label).

We additionally confirmed that genes identified in our array as being up-regulated by FGF treatment of trigeminal explants were indeed FGF-responsive by examining gene expression by in situ hybridization. *Foxi2*, *Cyp26C1* and *FGF8* were all predicted to be up-regulated by FGF from our array data, and this was confirmed by in situ hybridization following culture in medium containing FGF2 (Figure 3). Of these, our array identified *FGF8* and *Cyp26C1* as also being enriched in otic tissues, however whole mount analysis of their expression indicated that transcripts were largely absent from the otic placode and were instead strongly expressed in adjacent ectoderm. This highlights the difficulty in precisely dissecting otic placode tissue without contamination from neighboring ectodermal cells. The boundaries of the otic territory are not clearly demarcated at the placodal stages when dissection was performed and thus false positive results from non-otic tissues are likely to be present.

FGF signaling is instrumental during otic placode induction, and consequently several FGF-associated genes were detected in our otic enriched microarray dataset. The FGF ligands *FGF3, FGF8, FGF18,* and *FGF19* were represented, as was FGF Receptor-like 1 (*FGFRL1*), which lacks a tyrosine kinase domain and may act as an inhibitor of FGF signaling. Other FGF antagonists were also identified, including *Spry1* & *Spry2* [26] *SPRED1*, and *Sef* (IL17RD). The presence of multiple FGF pathway antagonists were consistent with the observation that FGF signaling has to be rapidly attenuated for correct differentiation of the otic placode [24], [26]. No FGF receptors were present in the otic-enriched list, although *FGFR1* was weakly enriched (1.3 fold) and *FGFR4* was not represented in the microarray probe sets. We also detected known downstream targets of FGF signaling, including the FGF/MAP kinase-activated Ets transcription factors *ETV3* and *ETV4* (*PEA3*) and genes associated with the MAP kinase signaling pathway, including the MAP kinase phosphatases *DUSP4* and *DUSP6*.

We were surprised to identify only 52 transcripts that were significantly enriched in the otic placode and also up-regulated in presumptive trigeminal ectoderm treated with FGF2 (Table 3). Although the 52 genes in this common list included known otic placode genes such as *Pax2*, *Eya2*, *Gbx2*, *Spry1* and *Spry2* [19], [26], [42], [46], [47], and many of the FGF-signaling associated genes mentioned above, a number of other known otic genes such as *Gata3*, *Has2* and *EphA4* were not up-regulated following FGF treatment. This raised the possibility that although FGF signaling may be necessary for the induction of the otic placode [10], [11], [12], [13], [24], [48], it may not be sufficient for the induction of many otic placode genes.

**Figure 4:**
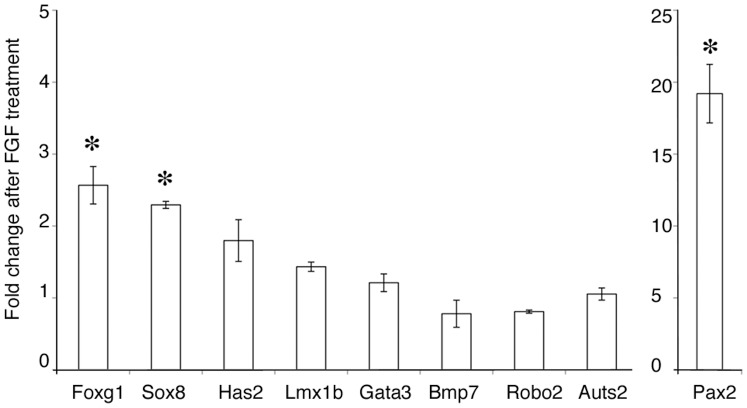
Validation of trigeminal ectoderm as an appropriate assay system for otic induction. To confirm that presumptive trigeminal placode tissue responded to FGF treatment in a similar manner presumptive otic placode tissue, we assayed the induction of nine otic placode genes in presumptive otic tissue (HH stage 8; a time where none of the genes have been induced) treated with FGF2 for 18 hours. Of the genes tested, only those identified in our array data as being both otic- and FGF-enriched (*Pax2*, *FoxG1*, & *Sox8*) were significantly up-regulated (* = p<0.05).

It is possible that presumptive trigeminal ectoderm is qualitatively different from presumptive otic ectoderm in its response to FGF2, and that this may explain the small number of otic genes that were also up-regulated when presumptive trigeminal ectoderm is treated with FGF2. To test this, we assayed the expression of 9 known otic genes by Q-PCR after culturing presumptive otic ectoderm in collagen gels in the presence or absence of 50 ng/ml FGF2 for 15 hours. Otic ectoderm was taken from chick embryos with 0–4 somites, before the onset of otic marker expression (ref. 19; Figure 4). Of the genes tested, only those identified in our array data as being both otic- and FGF-enriched (*Pax2*, *FoxG1*, & *Sox8*) were significantly up-regulated. We can therefore have confidence that our use of trigeminal ectoderm in FGF treatment experiments generates results broadly applicable to the otic placode.

### FGF Signaling through the MAP Kinase Pathway is Necessary to Induce the OEPD

**Figure 5:**
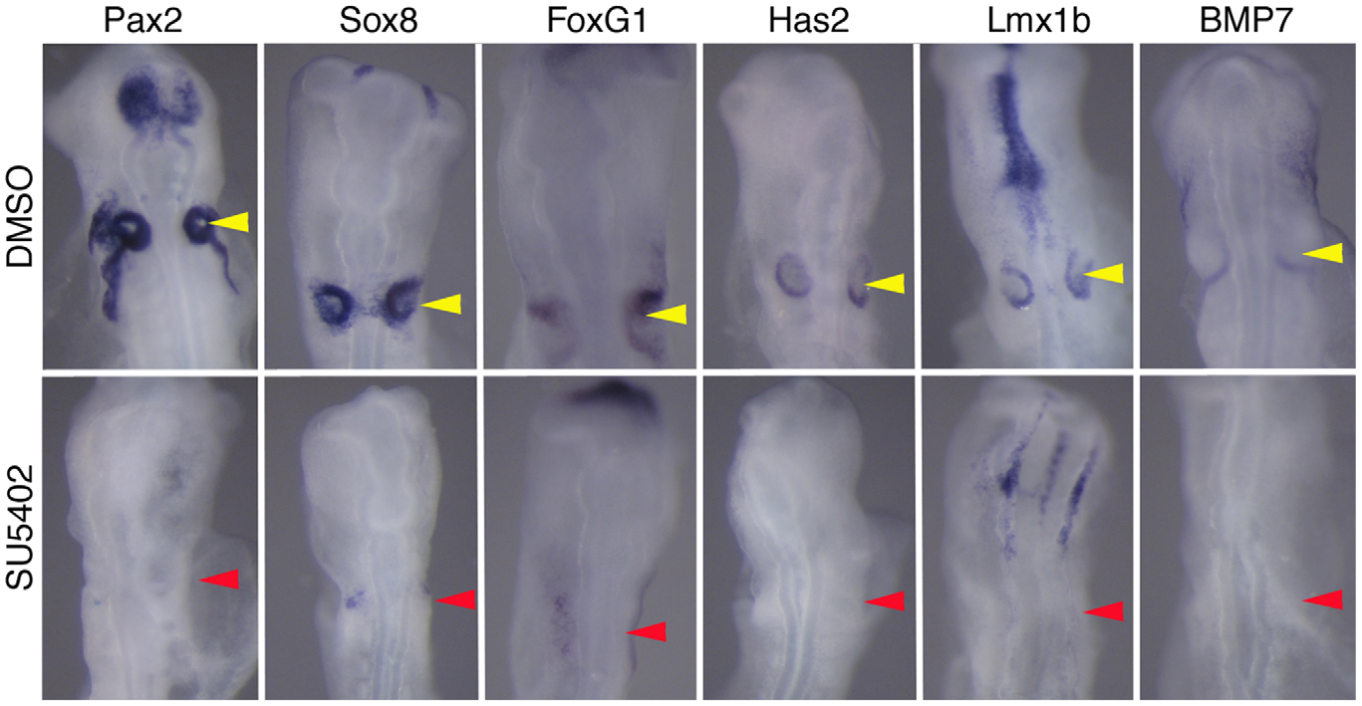
FGF signaling is necessary for the induction of otic placode genes. HH stage 8 chick embryos were cultured in the FGF receptor inhibitor SU5402 for 18 hours and assayed for the expression of otic placode genes. Out of 12 otic genes tested, all were significantly reduced in SU5402-treated embryos (Table 4). Six examples of down-regulated genes - (*Pax2*, *FoxG1*, *Sox8, Has2, Lmx1b* and *BMP7*) are shown in SU5402- or vehicle-treated embryos.

Our data suggested that FGF2 was only sufficient to induce a subset of otic genes in chick placodal ectoderm. We next tested whether FGF signaling was necessary for the induction of twelve otic placode genes (*Auts2, Bmp7, FoxG1, Gata3, Has2, Lmx1b, Musashi1, Otx2, Robo2, Il17rd*, *Sox8* and *Pax2*) by culturing whole embryos in the presence of 10 µM SU5402 for 18 hours to block FGFR signaling. In all cases, SU5402 either abolished or greatly reduced expression of each gene compared to DMSO vehicle controls (Table 5; Figure 5). Thus, although FGF signaling is *sufficient* to induce only a subset of otic placode genes, it is *necessary* for the expression of all genes tested.

**Table 5:** Inhibition of Otic Placode Gene Expression by the FGFR Inhibitor SU5402.

Gene	Control (DMSO)	SU5402 (10 µM)
*Auts2*	19/19	0/20
*Bmp7*	9/9	4/12
*Foxg1*	12/12	0/14
*Gata3*	19/19	1/20
*Has2*	9/9	0/11
*Lmx1b*	11/11	0/14
*Musashi1*	7/8	0/8
*Otx2*	7/8	0/12
*Pax2*	16/16	1/16
*Robo2*	17/17	2/25
*Sef (Il17rd)*	4/4	1/6
*Sox8*	12/12	0/13

Embryos were cultured in the FGFR inhibitor SU5402 or a DMSO vehicle control and then processed for in situ hybridization for 12 otic placode genes. Numbers refer to the number of embryos with in situ hybridization signal in the otic placode.

**Figure 6:**
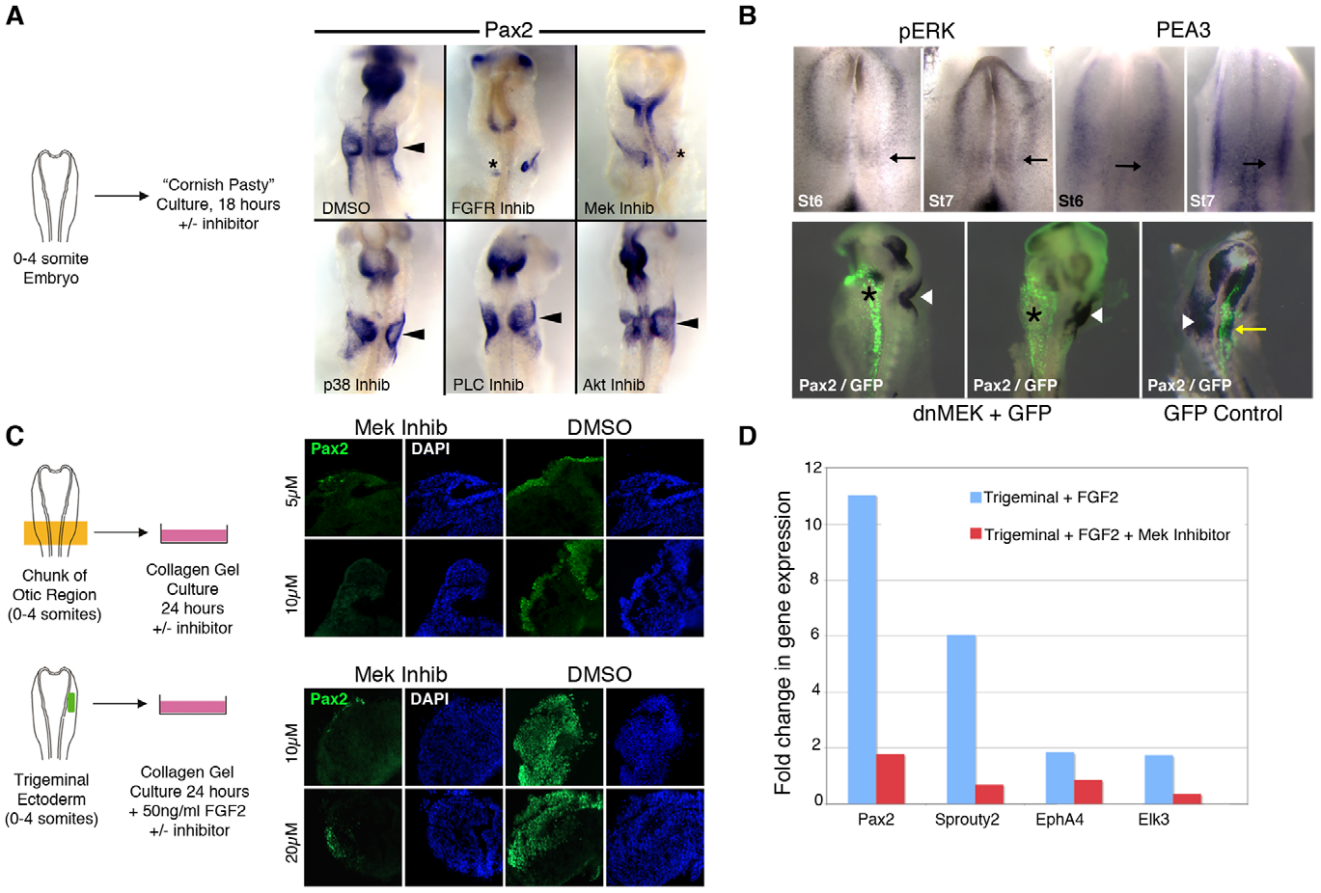
FGF2 regulates otic gene expression through the MAP kinase pathway. (A): HH stage 8 embryos were cultured for 18 hours in the presence of different FGF pathway inhibitors and assayed for expression of Pax2. Only embryos treated with the FGFR inhibitor SU5402 or the MEK kinase inhibitor U0126 showed decreased or abolished Pax2 expression. (B): FGF signaling is present in presumptive otic placodal ectoderm prior to the onset of otic placode genes. HH stage 6 and 7 embryos were either stained with antibodies to phosphorylated ERK kinase or processed for in situ hybridization with the FGF target gene PEA3. Electroporation of a dominant-negative MEK construct (together with a GFP reporter) down-regulates Pax2 expression in the otic region (asterisks) but not in control embryos (yellow arrow) or on the contralateral side of the embryo (arrowheads). (C): Inhibition of the MAP kinase pathway also blocks induction of Pax2 in cultured chunks of the developing otic region or in FGF-treated trigeminal level ectoderm. Embryo chunks or ectoderm pieces were sectioned and stained with antibodies to Pax2. The MEK inhibitor U0126 significantly reduced induction of Pax2 at both concentrations tested. (D): The MEK kinase inhibitor U0126 can also block the induction of other otic genes in addition to Pax2. Trigeminal level ectoderm was cultured with 50 ng/ml FGF2 in the presence or absence of 20 µM U0126. QPCR for the otic genes *Spry2, EphA4* and *Elk3* were all significantly reduced by U0126 treatment (p<0.05 in each case).

Activation of FGF receptors causes a downstream activation of a number of signaling cascades including the MAP kinase pathway, the PKC/PLCgamma pathway, PI3 Kinase/Akt pathway and the p38 MAP kinase pathway [49]. Our microarray data suggested that elements of the MAP kinase pathway might be up-regulated during otic placode induction and in response to FGF signaling. To test the necessity of different downstream signaling pathways from FGF receptors, we grew chick embryos at stage 7–8 (0–4 somite pairs) in floating cultures (“Cornish pasty” cultures; [32]) in the presence of different inhibitors of intracellular signaling pathways downstream from receptor tyrosine kinases and examined the expression of *Pax2* in the otic region (Figure 6A). As a positive control, we also included SU5402, a known inhibitor of FGF receptors [50]. SU5402 was able to block or greatly reduce the induction of *Pax2* in cultured chick embryos (22/24 embryos; 10 µM). The MAP kinase pathway inhibitor of Mek kinase, U0126 was also able to efficiently block or reduce *Pax2* induction (10/11 embryos; 50 µM). However, inhibitors of the PKC/PLCγ pathway (U73122; n = 10), PI3 Kinase/Akt pathway (LY294002; n = 11, Wortmannin; n = 9 or Akt Inhibitor IV; n = 3) and the p38 MAP kinase pathway (SB203580; n = 6) either resulted in no discernible effect on *Pax2* induction or else led to death of the embryos at higher concentrations tested.

We confirmed activation of the MAP kinase pathway at the early stages of otic placode induction by staining stage 6–7 chick embryos with antibodies against phosphorylated forms of the Erk MAP kinase and performing in situ hybridization for an Erk target, the Ets transcription family member *Pea3* (Figure 6B). In both cases, expression of phospho-Erk or *Pea3* could be observed in mesoderm underlying the future otic placode, and in a thin strip of ectoderm adjacent to the neural plate at the level of the otic placode. Electroporation of stage 6–7 chicken embryos with a dominant negative *Mek1* DNA construct (S218A and S222A *Mek1* double point mutant, [34]; 11/11 embryos) but not control DNA (n = 8) also efficiently blocked induction of *Pax2* (Figure 6B).

We also cultured chunks of heads from chick embryos with between 0–4 somite pairs in the presence of 5 or 10 µM U0126. Both concentrations of inhibitor significantly reduced the amount of PAX2 protein marking the otic placode (Figure 6C; 21/24 head chunks), whereas DMSO vehicle had no obvious effect. We have previously shown that presumptive trigeminal ectoderm can express PAX2 when cultured in collagen gels in the presence of FGF2. We repeated these experiments in the presence or absence of the MEK kinase inhibitor U0126. U0126 effectively blocked the expression of PAX2 protein in these cultures (Figure 6C; n = 17), but not control cultures (n = 9). U0126 also significantly reduced expression of *Pax2* and three other otic genes, *Spry2, EphA4* and *Elk3* when measured by Q-PCR (Figure 6D). Together these results suggest that activation of the MAP kinase pathway downstream of FGF receptors is necessary for the induction of at least some otic placode genes.

## Discussion

The induction of the otic placode and inner ear has been studied as a model of tissue induction for almost a century [1], [17]. In recent years, attention has focused on the role of FGF family members in inducing the otic placode [2]. In the present study we show that FGF signaling, although crucial in initiating otic placode formation, is in itself insufficient to activate the entire cascade of otic placode genes in cranial ectoderm. We used a microarray-based approach to first identify transcripts enriched in the otic placode, and then to establish which transcripts can be regulated by FGF signaling in culture. We assessed the quality of our array data by comparison with previously characterized otic and FGF-associated genes, and by our own in situ hybridization experiments. Our findings indicate that surprisingly few otic genes are up-regulated by FGF treatment alone, although FGF signaling is necessary for the expression of all otic genes examined.

We used a trigeminal-fated region of the preplacodal domain for our FGF treatment experiments, as this eliminates the prospect of unwanted contamination of our sample with otic genes. The induction of many otic markers has confirmed that this is a reasonable approach, as has previous grafting experiments that demonstrate that trigeminal level ectoderm from early somite stages is competent to undergo limited otic morphogenesis [19]. Our trigeminal data was also consistent with data obtained by QPCR using presumptive otic placode tissue; of the genes tested, only those up-regulated by FGF in trigeminal tissue were also up-regulated by FGF in the otic samples. These experiments operate under the assumption that the pre-placodal field is effectively uniform, and that an otic placode can be effectively induced from different axial levels. This is true in amphibians, where rotation of the placodal field can result in anteriorly located otocysts [51]. Expression analysis also generally supports uniformity of the preplacodal domain [6], [52], although a Six1 enhancer specific for the rostral half of the preplacodal region has been recently identified [53].

The genes identified by our arrays as being otic-enriched correlate well with a similar experiment performed by Paxton and colleagues [54]. Using Agilent chicken genome arrays, they identified genes expressed in stage 7 chicken embryos in the pre-otic region by comparing its expression profile with more rostral tissues. Although these data are not ectoderm-specific, and are taken from slightly younger embryos, broad comparisons with our results can be made. Of the select genes presented in Table 1 of Paxton et al., [54], 8 are also found in our otic enriched set (*Fgf3, Fgf19, Fzd8, Hes5, Hes6, Jag1, Gbx2,* & *Meox1*). Notable among these factors are the presence of 3 Notch signaling associated genes: the Notch ligand *Jag1*, and the downstream Notch targets *Hes5*, and *Hes6*. Our array additionally identified *Dll1* and *Hey1* as otic enriched transcripts. This is consistent with previous reports highlighting the importance of Notch signals in early otic formation [55], [56].

A second array-based experiment has recently examined otic placode genes downregulated in *Fgf3* and *Fgf10* conditional null mice [29]. The number of genes identified as differentially expressed in otic placodal ectoderm was low (28 genes down-regulated in *Fgf3*/*Fgf10* double null embryos), and several known otic placode genes detected in our experiments were not picked up by the mouse arrays (for example *Pax2*, *Gbx2*, *Sprouty1/2*). Species differences may account for some of the observed differences, as mouse and chicken are known to occasionally use different but related genes during otic development [57]. Urness and colleagues also attribute the relatively small number of FGF-dependent genes in their study to the technical difficulties of isolating the young otic placode from mouse embryos. Nevertheless, in both mouse and chicken, our data and that of Urness show roles for members of *Sox*, *Zic*, *ZNF*, *GPR*, *Kif*, *Sic, SPINK*, and *DUSP* family genes, in addition to *FoxG1* and *Has2*.

Our validation of the array results by gene expression analysis and database searches suggest that our data is robust. However our microarray approach has some inherent limitations that should be considered when interpreting these data. First, although comprehensive, the transcripts represented in our microarrays are not a complete list of all chicken genes. It is unavoidable that some otic associated and FGF-sensitive genes will be overlooked, as they are simply absent from the array. Our in situ analysis also identified some genes that were wrongly identified as otic enriched, due to being strongly expressed in neighboring tissues. Small variations in the precise region dissected between embryos are difficult to eliminate and thus such false positive results are an unavoidable consequence of our methodology.

We conclude that most otic genes are not FGF-responsive, and that most FGF-responsive genes from trigeminal tissue are not otic genes. Interestingly, although only 15% (52/344) of total otic enriched transcripts were induced by FGF, among the most highly enriched samples the ratio was higher: 55% (11/20) of the top 20 otic genes were up-regulated by FGF. In contrast to FGF addition experiments, chemical blockade of FGF signaling by SU5402 effectively blocked expression of all examined otic genes. Thus, although FGF signaling is not sufficient to drive the entire otic program, it is clearly necessary for otic placode induction, indeed its continued action is inhibitory to full otic formation [24]. This result supports a model in which all subsequent otic inductive events are dependent on an initial FGF step. We previously proposed that the expression of preplacodal marker genes may represent a first step in otic induction, as the onset of preplacodal gene expression correlates with competence to respond to FGF treatment [30]. In this study, we used 1–4 ss trigeminal ectoderm in our cultures, which by this stage already expresses preplacodal markers. Therefore, the subsequent actions of FGF signaling on this tissue may perhaps then be considered as the second such induction step, driving preplacodal ectoderm to an “early otic placode” state, from which further (as yet unidentified) signaling is required to continue inner ear development. We hypothesized that Wnt may be the missing signaling link in otic placode formation, as Wnt has previously been demonstrated to help control otic placode specification [16], [24], [28]. Indeed the Wnt receptors *Fzd7*, and *Fzd8* were induced in our FGF treatment experiment. Preliminary experiments however, indicated that activation of the Wnt pathway (using the Gsk3 inhibitor LiCl) in addition to FGF in our cultures of trigeminal ectoderm does not result in activation of the complete otic pathway, although it does significantly increase expression of *Pax2* (data not shown), consistent with observations in the zebrafish [12].

Our data clearly shows that the MAP kinase pathway is likely the principal intracellular signaling effector of FGF signaling during early otic placode development. During otic placode invagination, which occurs slightly later in embryogenesis, the PLC-gamma pathway has been implicated as being important for cell shape changes in response to FGF signaling [58]. Therefore, it is possible that the same receptor pathway could be affecting different aspects of otic development at different times via distinct mechanisms. Importantly, our study has confirmed that FGF signals regulate some, but not all otic gene expression and that additional signals that may regulate other aspects of otic induction remain to be identified. We now approach a time when all of the necessary molecular components of otic induction will be known, but a significant challenge remains in piecing together the complex interplay that directs otic development. Recent progress has been made in the assembly of placode-associated gene regulatory networks [59]. Region-specific expression profiles of different placodes such as our present study will provide the raw materials from which similar gene networks can be built and extended.

## Supporting Information

Table S1 **List of all otic-enriched genes.**
(XLSX)

Table S2 **List of all FGF up-regulated genes.**
(XLSX)

Table S3 **List of all otic-enriched genes also up-regulated by FGF.**
(XLSX)

Table S4 **List of primers for making in situ probes.** All reverse primers contain a T7 polymerase site (GGATCCTAATACGACTCACTATAGGGAG).
(DOCX)

Table S5 **List of primers for Q-PCR.**
(DOCX)
