# Supplementary material for: Analysis of FGF-Dependent and FGF-Independent Pathways in Otic Placode Induction
Source: PLoS One. 2013 Jan 23;8(1):e55011. doi: 10.1371/journal.pone.0055011 (PMC3552847; doi:10.1371/journal.pone.0055011)
Supplement: Table S4 — List of primers for making in situ probes. All reverse primers contain a T7 polymerase site (GGATCCTAATACGACTCACTATAGGGAG). (DOCX) [file pone.0055011.s004.docx]

**Table S4: Primers for making in situ probes**

| **Gene** | **Accession No.** | **Primer Sequences** | **Size (bp)** |
| --- | --- | --- | --- |
| *Foxg1* | NM_205193 | F: GTTCAGCTACAACGCGCTCATCAT | 981 |
|  |  | R: GGATCCTAATACGACTCACTATAGGGAGTCATCATTTACAACGCGAACGTGTG |  |
| *Has2* | NM_204806 | F: CAGTTAGTGGGCCTGATAAAGTCTTCC | 825 |
|  |  | R: GGATCCTAATACGACTCACTATAGGGAGAAAGAGAAGTTCATAATTAGGTTGCAAGGG |  |
| *Sox8* | AF228664 | F: CACGCCGCCCACGA | 875 |
|  |  | R: GGATCCTAATACGACTCACTATAGGGAGTTGGAGAGTTTCAAAGCAAGGG |  |

All reverse primers contain a T7 polymerase site (GGATCCTAATACGACTCACTATAGGGAG)
