# Supplementary material for: Analysis of FGF-Dependent and FGF-Independent Pathways in Otic Placode Induction
Source: PLoS One. 2013 Jan 23;8(1):e55011. doi: 10.1371/journal.pone.0055011 (PMC3552847; doi:10.1371/journal.pone.0055011)
Supplement: Table S5 — List of primers for Q-PCR. (DOCX) [file pone.0055011.s005.docx]

**Table S5: List of Primers for Q-PCR**

| **Gene** | **Accession Number** | **Primers** | **Product Size (bp)** |
| --- | --- | --- | --- |
| *Auts2* | XM_415729 | F: GAACAGGCTACTTTTGCGACAGCG | 137 |
|  |  | R: AGGGTCGGCATTTTCAGCGAGC |  |
| *Bmp7* | AF205877.1 | F: **TGGTCATGAGCTTCGTCAAC** | 102 |
|  |  | R: **CCTCTGGGATTCTGGAGAGA** |  |
| *Elk3* | NM_001030749.1 | F: TGCCAAGTGCCTTCTACCACTTCT | 120 |
|  |  | R: ACAAGCAGCAGACATTTGCAGTCC |  |
| *EphA4* | NM_204781.1 | F: GTTTGCAATGTGATGGAGCCCAGT | 112 |
|  |  | R: TGCAGTCTCTCAGCGTGAACTTGA |  |
| *Foxg1* | NM_205193 | F: CAACGGCATCTACGAGTTCA | 116 |
|  |  | R: GCACCTTGACGAAGCACTTA |  |
| *Gata3* | NM_001008444.1 | F: ACTTACCCACCGTATGTCCCCG | 135 |
|  |  | R: TACACACTCCCTGCCTTCTGTGC |  |
| *GAPDH* | NM_204305.1 | F: GAGGGTAGTGAAGGCTGCTG | 113 |
|  |  | R: CATCAAAGGTGGAGGAATGG |  |
| *Has2* | NM_204806 | F: AAGATCCAATGGTTGGAGGA | 79 |
|  |  | R: CTGCTCAGAAAGGAGATCCAG |  |
| *Lmx1b* | NM_205358 | F: ATGGAAGATCACCCGCTGCG | 61 |
|  |  | R: ACTCCGACCCCAGTAACACTCC |  |
| *Pax2* | NM_204793.1 | F: ATCTGCGACAACGACACGGTCC | 138 |
|  |  | R: GCTGGGCACGATAGTATGTCCTGG |  |
| *Robo2* | AY600957.1 | F: GTCCGCTGGAAGAAGGATG | 81 |
|  |  | R: CTTCTTGATGCGCAAAGTGT |  |
| *S16* | [XM_416113.2](http://www.ncbi.nlm.nih.gov/nuccore/XM_416113.2) | F: AATGATTGAGCCTAGGACTTTGCA | 81 |
|  |  | R: TCAACACCAGCAAATCTCTCCTT |  |
| *Sox8* | AF228664 | F: GCAAGCTCTGGCGTTTGTTA | 61 |
|  |  | R: CGCTCAGCTTCTTCCACAA |  |
| *Spry2* | NM_204800.1 | F: AAGACTTGGGAGCACACAGCTACA | 149 |
|  |  |  |  |
